# Extended gene panel testing in lobular breast cancer

**DOI:** 10.1007/s10689-021-00241-5

**Published:** 2021-03-25

**Authors:** Elke M. van Veen, D. Gareth Evans, Elaine F. Harkness, Helen J. Byers, Jamie M. Ellingford, Emma R. Woodward, Naomi L. Bowers, Andrew J. Wallace, Sacha J. Howell, Anthony Howell, Fiona Lalloo, William G. Newman, Miriam J. Smith

**Affiliations:** 1 Manchester Centre for Genomic Medicine, Manchester University Hospitals NHS Foundation Trust, Manchester, UK; 2 Division of Evolution and Genomic Sciences, School of Biological Sciences, Faculty of Biology, Medicine and Health, University of Manchester, Manchester Academic Health Science Centre, Manchester, UK; 3 Prevent Breast Cancer Centre, Wythenshawe Hospital Manchester Universities Foundation Trust, Wythenshawe, Manchester, UK; 4 Manchester Breast Centre, The Christie NHS Foundation Trust, Wilmslow Road, Manchester, UK; 5 Division of Informatics, Imaging and Data Sciences, Faculty of Biology, Medicine and Health, University of Manchester, Manchester Academic Health Science Centre, Manchester, UK; 6 Division of Cancer Sciences, Faculty of Biology, Medicine and Health, University of Manchester, Manchester Academic Health Science Centre, Manchester, UK

**Keywords:** Lobular breast cancer, Panel testing, Genetics, *ATM*

## Abstract

*Purpose*: Lobular breast cancer (LBC) accounts for ~ 15% of breast cancer. Here, we studied the frequency of pathogenic germline variants (PGVs) in an extended panel of genes in women affected with LBC. *Methods*: 302 women with LBC and 1567 without breast cancer were tested for *BRCA1/2* PGVs. A subset of 134 LBC affected women who tested negative for *BRCA1/2* PGVs underwent extended screening, including: *ATM, CDH1, CHEK2, NBN, PALB2, PTEN, RAD50, RAD51D,* and *TP53.* *Results*: 35 PGVs were identified in the group with LBC, of which 22 were in *BRCA1/2*. Ten actionable PGVs were identified in additional genes (*ATM*(4), *CDH1*(1), *CHEK2*(1), *PALB2*(2) and *TP53*(2)). Overall, PGVs in three genes conferred a significant increased risk for LBC. Odds ratios (ORs) were: *BRCA1*: OR = 13.17 (95%CI 2.83–66.38; P = 0.0017), *BRCA2*: OR = 10.33 (95%CI 4.58–23.95; P < 0.0001); and *ATM:* OR = 8.01 (95%CI 2.52–29.92; P = 0.0053). We did not detect an increased risk of LBC for *PALB2, CDH1* or *CHEK2*. *Conclusion*: The overall PGV detection rate was 11.59%, with similar rates of *BRCA1/2* (7.28%) PGVs as for other actionable PGVs (7.46%), indicating a benefit for extended panel genetic testing in LBC. We also report a previously unrecognised association of pathogenic variants in *ATM* with LBC.

**Supplementary Information:**

The online version contains supplementary material available at 10.1007/s10689-021-00241-5.

## Introduction

Invasive lobular cancer (ILC) is the most common special histological subtype of breast cancer representing 10–15% of cases overall. ILC is associated with a higher risk of inheritance than invasive ductal cancers (IDC) which are also termed 'no special type' and represent 70% of cases overall [1, 2]. Pathogenic germline variants (PGVs) in one of the known high-risk breast cancer susceptibility genes e.g. *BRCA1*, *BRCA2*, *PALB2* confer a high risk of developing breast cancer and are enriched in familial cases. Notably, lobular breast cancer (LBC) is less common as a proportion of breast cancers in women with *BRCA1* PGVs compared to women without *BRCA1* PGVs [3–6] As the main pathway to breast cancer is from basal progenitor cells leading to the predominant ductal triple negative breast cancer, this route excludes the predominantly estrogen receptor positive lobular cancer. However, individuals with *BRCA2* PGVs have a similar proportion of LBC to women without *BRCA2* PGVs. Familial breast cancer not associated with variants in *BRCA1* or *BRCA2* is more likely to be lobular than those with a *BRCA1* or *BRCA2* variant [7, 8]. In our first pathology update of the Manchester Scoring system, 11% of familial breast cancers that tested negative for *BRCA1/2* were lobular, but lobular cancer was present in only 1.6% of index *BRCA1* cases [8].

A recent increase in the use of multi-gene panels to screen for breast cancer-associated gene variants beyond *BRCA1* and *BRCA2* has started to identify PGVs in additional genes. Several studies have reported the association for the diffuse gastric cancer predisposition gene, *CDH1*, in lobular, rather than ductal breast cancer [9–12]. Petridis et al. screened six genes for an association with LBC and found that variants in *BRCA2*, *CHEK2*, and *PALB2* in addition to *CDH1* were all enriched in women with lobular compared to ductal cancer [5].

Only PGVs in the *CDH1* gene have been convincingly shown to cause a specific high risk of LBC with lifetime risks of about 40% in women [13, 14]. However, *CDH1* germline PGVs are very rare with a population incidence of 1 in 10–100,000 [15]. Indeed, a study of 1434 women with lobular breast cancer only found five (0.35%) with a *CDH1* PGV [5]. As such there are still many individuals with LBC and a family history of breast cancer in whom no PGV has been identified.

Here, we present an analysis of the frequency of detectable pathogenic variants including those in an extended panel of nine non-*BRCA1/2* genes in a cohort of 302 women with LBC.

## Methods

### Patient materials

Women were eligible for this study if they had a histologically confirmed diagnosis of LBC (lobular invasive lobular carcinoma). A total of 302 women affected with LBC were included in the study. Two-hundred and sixty four of these women were seen at the Manchester Centre for Genomic Medicine (MCGM) and 259 (98.1%) of these had a family history of breast or ovarian cancer or a second primary breast/ovarian cancer. The first families ascertained through MCGM were from 1990, although testing for *BRCA1/2* only started in 1996. A further 38 women took part in the population-based study, Predicting the Risk Of Cancer At Screening (PROCAS), held in Greater Manchester [16]. Additionally, 1567 women without a breast cancer diagnosis at entry (aged 46–73 years) who were also recruited to the PROCAS study were included as controls. To obtain a population average dataset we included 124 women who subsequently developed breast cancer to provide an ~ 8% population risk of breast cancer at a median last age of follow up of 69 years. Clinical or research consent was given for extended testing of breast cancer associated genes (approval from the North Manchester Research Ethics Committee (reference 09/H1008/81(PROCAS) and 08/H1006/77)).

### Genetic screening

For women that were seen at the MCGM, DNA was extracted from lymphocytes and all these samples were initially sequenced for PGVs in *BRCA1/2* by a combination of Next Generation Sequencing and MLPA. A subset of 134 of the patients that did not harbour a PGV in either of those genes underwent extended testing. All women recruited through PROCAS provided a saliva sample for DNA extraction. They underwent panel testing as part of the Breast Cancer Risk after Diagnostic Gene Sequencing (BRIDGES) study [17]. The BRIDGES study performed sequencing of 33 genes (*AKT1, ATM, BARD1, BRCA1, BRCA2, BRIP1, CDH1, CHEK2, EPCAM, FAM175A, FANCC, FANCM, GEN1, MEN1, MRE11A, MSH2, MSH6, MUTYH, NBN, NF1, PALB2, PIK3CA, PMS2, PPM1D, PTEN, RAD50, RAD51C, RAD51D, RECQL, RINT1, STK11, TP53* and *XRCC2*). Forty-six women underwent clinical panel testing (the panel included: *ATM, BAP1, BRCA1, BRCA2, CDH1, CHEK2, MLH1, NBN, PALB2, PTEN, RAD50, RAD51D, RECQL, TP53*, and for 20 of these women *BARD1, MSH6, MSH2* and *PMS2* were also included) and 50 women underwent targeted exome sequencing through the Beijing Genomics Institute (BGI), Shenzhen, Guangdong, China. A minimum of nine genes were screened in each patient that underwent extended testing. These genes were: *ATM, CDH1, CHEK2, NBN, PALB2, PTEN, RAD50, RAD51D,* and *TP53* (Supplemental Fig. 1).

Samples that underwent panel testing though BRIDGES did not undergo copy number analysis. The samples that underwent targeted exome sequencing only had copy number testing for *BRCA1/2*.

Variants were classified according to the ACMG guidelines [18]. All identified pathogenic variants were confirmed through the clinical diagnostics laboratory. Only variants that were classified as ‘likely pathogenic’ or ‘pathogenic’ are reported here.

Tumour pathology information was obtained for each case through hospital records, and cancer registries as previously described [19]. The probability of a *BRCA1/2* PGV was determined using the Manchester score (MS) for each affected individual [20]. Genes were considered actionable for breast cancer if they had published data confirming at least a twofold relative risk of breast cancer [21]. Associations between LBC and PGVs in the screened genes were calculated by Fisher’s exact test using GraphPad Prism 8.

## Results

### Pathogenic variants

A total of 302 women affected with invasive LBC were initially screened for PGVs in *BRCA1/2*. Within this group 22 (7.28%) PGVs were identified in *BRCA1/2* (5 in *BRCA1* (1.66%) and 17 in *BRCA2* (5.63%)). Of the 280 women who tested negative for *BRCA1/2* PGVs, 134 (47.9%) had sufficient DNA available for extended panel testing of at least *ATM, CDH1, CHEK2, NBN, PALB2, PTEN, RAD50, RAD51D,* and *TP53*. This resulted in the detection of an additional 13 PGVs of which 10 (7.46%) could be considered to be actionable with at least a confirmed two fold increased breast cancer risk [21] (4 in *ATM*, 1 in *CDH1,* 1 in *CHEK2,* 2 in *PALB2,* and 2 in *TP53*) (Table 1). Additional PGVs genes with a less clear association with breast cancer were found in *MSH6, RAD50,* and *NBN.* Thus, in total, PGVs were detected in 35 individuals (11.59%). *MSH6* was not assayed on every clinical gene panel and was therefore screened in 108 of the 134 cases. Twenty-five women with lobular carcinoma *in situ* were also screened for PGVs, but none of them carried a PGV.

**Table 1:** Pathogenic variants identified

Individual	BC age at diagnosis (years)	Receptor status	Gene	HGVS Annotation	Consequence	MS
1	46	ER +	*ATM*	c.3802deIG; p.(Val1268*)	Truncating	≥ 20
2	63	ER + /HER2-	*ATM*	c.4741delA; p.(Ile1581Serfs*20)	Truncating	< 20
3	49	ER + /HER2-	*ATM*	c.5155delA; p.(Asn1719Ilefs*5)	Truncating	≥ 20
4	49	ER +	*ATM*	c.8494C > T; p.(Arg2832Cys)	Missense	< 20
5	46	ER + /HER2-	*BRCA1*	c.1961delA; p.(Lys654Serfs*47)	Truncating	≥ 20
6	43	ER-	*BRCA1*	c.4106delC; p.(Ala1369Aspfs*24)	Truncating	< 20
7	29	ER +	*BRCA1*	c.68_69delAG; p.(Glu23Valfs*17)	Truncating	≥ 20
8	36	Unknown	*BRCA1*	Deletion exon 1–2	CNV	≥ 20
9	51	ER +	*BRCA1*	c.547 + 1G > T; p.?	Splice variant	< 20
10	33	ER-	*BRCA2*	c.1929delG; (p.Arg645fs*15)	Truncating	≥ 20
11	57	ER + /HER2-	*BRCA2*	c.1929delG; p.(Arg645Glufs*15)	Truncating	< 20
12	49	ER +	*BRCA2*	c.4478_4481delAAAG; p.(Glu1493Valfs*10)	Truncating	< 20
13	49	ER + /HER2-	*BRCA2*	c.470_474delAGTCA; p.(Lys157Serfs*24)	Truncating	≥ 20
14	28	ER +	*BRCA2*	c.5303_5304delTT; p.(Leu1768Argfs*5)	Truncating	< 20
15	45	Unknown	*BRCA2*	c.5682C > G; p.(Tyr1894*)	Truncating	≥ 20
16	39	ER + /HER2-	*BRCA2*	c.5682C > G; p.(Tyrl894*)	Truncating	< 20
17	42	ER +	*BRCA2*	c.5909C > A; p.(Ser1970*)	Truncating	< 20
18	33	ER +	*BRCA2*	c.6275_6276delTT; p.(Leu2092Profs*7)	Truncating	≥ 20
19	60	ER + /HER2-	*BRCA2*	c.6275-6276delTT; p.(Leu2092Profs*7)	Truncating	≥ 20
20	45	ER +	*BRCA2*	c.6602delC; p.(Ser2201Leufs*5)	Truncating	< 20
21	33	ER +	*BRCA2*	c.695dupA; p.(Tyr232*)	Truncating	< 20
22	46	ER + /HER2-	*BRCA2*	c.7480C > T; p.(Arg2494*)	Truncating	≥ 20
23	38	ER +	*BRCA2*	c.7884dupA; p.(Trp2629Metfs*12)	Truncating	< 20
24	46	ER + /HER2-	*BRCA2*	c.8170_8190delinsCTAACTTA; p.(Gly2724Leufs*5)	Truncating	≥ 20
25	49	Unknown	*BRCA2*	c.8575delC; p.(Gln2859Lysfs*4)	Truncating	≥ 20
26	38	ER + /HER2 +	*BRCA2*	c.9157delG; p.(Glu3053Serfs*9)	Truncating	< 20
27	50	ER +	*CDH1*	Deletion exon 1–2	CNV	< 20
28	57	ER +	*CHEK2*	c.1100delC; p.(Thr367Metfs*15)	Truncating	< 20
29	62	ER-/HER2 +	*MSH6*	c.2910G > A; p.Trp970*	Truncating	< 20
30	53	Unknown	*NBN*	c.156_157delTT; p.(Ser53Cysfs*9)	Truncating	< 20
31	45	ER + /HER2-	*PALB2*	c.196C > T; p.(Gln66*)	Truncating	< 20
32	38	Unknown	*PALB2*	c.3549C > G; p.(Tyr1183*)	Truncating	≥ 20
33	64	ER + /HER2-	*RAD50*	c.3G > A; p.(Met1?)	Truncating	< 20
34	46	Unknown	*TP53*	c.538G > A; p.(Glu180Lys)	Missense	≥ 20
35	21	ER +	*TP53*	c.949C > T; p.(Gln317*)	Truncating	< 20

*BC* Breast cancer, *MS* Manchester score, *CNV* copy number variant, *HGVS* Human Genome Variation Society

In the control group of 1567 women, a total of 36 PGVs (2.30%) were detected. A first degree family history of breast cancer was present in 11.9% of PROCAS controls who provided a saliva DNA (and were analysed in BRIDGES) compared to 11.3% of PROCAS controls who did not provide a DNA sample. This indicates that there is no bias towards family history in providing DNA samples. There were eleven PGVs in *BRCA1/2* (2 in *BRCA1* and 9 in *BRCA2*), as well as six in *ATM*, six in *CHEK2*, three in *PALB2,* three in *NBN*, one in *MSH6,* and six in *RAD50*.

Odds ratios (ORs) for each gene are presented in Table 2. The ORs for *BRCA2* (OR = 10.33 (95%CI 4.58–23.95; P < 0.0001)), *BRCA1* (OR = 13.17 (95%CI 2.83–66.38; P = 0.0017)), and *ATM* (OR = 8.01 95%CI 2.52–29.92; P = 0.0053) confirmed elevated LBC risks with the lower 95% confidence intervals (CI) above twofold. We were unable to confirm this increase in LBC risk for *PALB2* and *CHEK2*. *CDH1* was non assessable due to absence of a reliable population incidence and the identification of only one case, previously reported [22]. All patients were also tested for *CHEK2* c.1100delC. We only identified one woman with LBC and six controls with this PGV (OR = 0.86; 95%CI 0.08–5.23; P  ≥ 0.9999).

**Table 2:** Association of pathogenic variants in women with lobular breast cancer

Truncating OR	Total	*BRCA1*	*BRCA2*	*TP53*	*PALB2*	*ATM*	*CHEK2*	*CDH1*	*NBN*	*RAD50*	*MSH6*^b^
PROCAS controls	1567	2	9	0	3	6	6	0	3	6	1
%		0.13%	0.57%	0.00%	0.19%	0.38%	0.38%	0.00%	0.19%	0.38%	0.06%
Lobular cases excluding LCIS	302/134^a^	5	17	2	2	4	1	1	1	1	1
%		1.66%	5.63%	1.42%	1.49%	2.99%	0.75%	0.75%	0.75%	0.75%	0.93%
OR		**13.17**	**10.33**		7.90	**8.01**	1.96		3.92	1.96	14.64
95% CI		**2.83–66.38**	**4.58–23.95**		1.38–38.87	**2.52–29.92**	0.17–11.94		0.30- 26.39	0.17–11.94	0.76–278.1
P-value		**0.0017**	** < 0.0001**		0.0526	**0.0053**	0.4575		0.2800	0.4575	0.1248

Bold values indicates *P* < 0.05

^a^Total of women tested for BRCA1/2 variants is 302, and for extended panel of genes 134

^b^MSH6 was only tested in 108 women

### *ATM* variant segregation

One of the individuals with an *ATM* PGV did not have a family history of breast cancer. For only one family, an additional family member was available to perform segregation analysis. This family member was affected with ductal breast cancer rather than lobular breast cancer and also carried the same *ATM* PGV. The other two families also had mixed lobular and ductal pathologies.

### Receptor status

Of the 302 women, receptor status was known for 220 (72.82%). The majority were estrogen receptor (ER) positive (214 cases, 97.27%). Of the ER positive cases, HER2 receptor status was also known in 137 cases and, of these, 128 (93.43%) were HER2-negative (Table 3).

**Table 3:** Distribution of pathogenic variants according to Manchester score and receptor status

Manchester score	All	PGVs	%	*BRCA1*	*BRCA2*	*% BRCA1/2*	*TP53*	*PALB2*	*ATM*	*CHEK2*	*CDH1*	*NBN*	*RAD50*	*MSH6*
MS < 15	171	11	6.43	2	4	3.51		1	1		1		1	1
MS ≥ 15 < 20	61	9	14.75	0	5	8.20	1		1	1		1		
MS < 20	232	20	8.62	2	9	4.74	1	1	2	1	1	1	1	1
MS ≥ 20	70	15	21.43	3	8	15.71	1	1	2					
Total	302	35	11.59	5	17	7.28	2	2	4	1	1	1	1	1
Receptor status														
ER-	4	2	50.00	1	1	50.00								
ER-/HER2 +	1	1	100.00			0.00								1
ER-/HER2-	1	0	0.00			0.00								
ER +	79	14	17.72	2	7	11.39	1		2	1	1			
ER + /HER2 +	8	1	12.50		1	12.50								
ER + /HER2-	127	11	8.66	1	6	5.51		1	2				1	
Unknown	95	6	7.32	1	2	3.66	1	1				1		
Total	328	35	11.59	5	17	7.28	2	2	4	1	1	1	1	1

*MS* Manchester score, *PGVs* pathogenic germline variants

In three of the only six ER negative cases, a PGV was present (*BRCA1*(1); *BRCA2*(1); *MSH6*(1)). The majority of PGVs were identified in women with ER positive (14 PGVs: *BRCA1*(2); *BRCA2*(7); *TP53*(1); *ATM*(2); *CDH1*(1); *CHEK2*(1)) or ER positive/HER2- negative receptor status (11 PGVs: *BRCA1*(1); *BRCA2*(6); *PALB2*(1); *ATM*(2); *RAD50*(1)). One PGV was found in a woman with ER positive/HER2 positive tumour receptor status (*BRCA2*) and six PGVs (*BRCA1*(1); *BRCA2*(2); *TP53*(1); *PALB2*(1); *NBN*(1)) were found in women whose receptor status was unknown (Table 3).

### Manchester score

In order to assess the probability of PGVs in *BRCA1/2*, MS was determined for all affected women. The majority of women had a MS < 20 (232/302) and a detection rate of 4.74%. The detection rate of *BRCA1/2* PGVs in women with a MS > 20 was 15.71% (Table 3).

Only 43 of the 302 (14.23%) women did not have a family history of breast cancer and four of these carried an actionable PGV (1 in *BRCA1,* 1 in *BRCA2* and 1 in *TP53* and 1 in *ATM*). The *BRCA1* and *BRCA2* PGVs were identified in women diagnosed with a first primary lobular cancer who then developed a second primary breast or ovarian cancer, respectively, whereas the *TP53* PGV was identified in a woman with a first primary lobular breast cancer at age 21 who then developed bilateral disease.

## Discussion

In this cohort of 302 women diagnosed with LBC, we identified a PGV in 11.59%. Interestingly, the detection rate of PGVs beyond *BRCA1/2* was 7.46%, which is similar to the *BRCA1/2* detection rate (7.28%). Although the overall rate was low, the comparable rates of detection between *BRCA1/2* and non-*BRCA1/2* variants implies that extended testing may be particularly beneficial in women with LBC and a family history of breast cancer.

We identified four PGVs in *ATM*, which is equal to the number of PGVs detected in *BRCA1* even though *ATM* was screened in less than half the individuals where *BRCA1* was tested. The OR of 8.01 and two fold increased risk at the lower end of the confidence interval supports an association of *ATM* PGVs with LBC, which is consistent with Lu et al. [23] This group identified six PGVs in *ATM* in 369 (1.63%) patients with LBC (OR = 3.50; 95%CI 1.10–9.73), although this is a lower frequency than in our study (4/134 (2.99%)).

In line with previous observations, the four *ATM* PGVs identified in this study were found in women with ER + (/HER-) tumour characteristics [24, 25], which is also the most common tumour type seen in LBC [26].

In this study, we found as many PGVs in *ATM* as in *PALB2* and *CHEK2* combined. The recent large study by Petridis et al. [5] found that *BRCA2, PALB2* and *CHEK2* PGVs were the most prevalent in women affected with LBC [5]. However, *ATM* was not investigated in the Petridis study. A recent large American-based study of germline genetic testing criteria in 3907 women with breast cancer, identified 43 *ATM* variants, but did not distinguish between women with ductal and lobular cancers [27].

*CDH1* is known to predispose to diffuse gastric cancer and has more recently been associated with LBC [5, 12]. In our cohort, we only identified one *CDH1* PGV in a LBC family and there was no history of gastric cancer in this family. Another recent study reported an association with *MSH6* (PGV identified in 7 of 590 patients) [23], although this was questioned as being due to potential sequencing errors [28]. We were not able to evaluate this fully as *MSH6* variants were screened in only 80.6% of the samples tested and our study was not powered to refute this association.

We identified two cases with a *TP53* PGV. This is in contrast to Petridis et al. who did not identify any *TP53* PGVs in an unselected series of 1434 lobular cancers [5]. A lack of association with lobular cancer and germline *TP53* was also suggested by Ditchi et al. in 2019 [6] but this is based on one case in only 57 carriers and the 95% CI do not exclude a 10% rate. One of our women was diagnosed with very early onset, bilateral breast cancer (the contralateral tumour was reported as invasive ductal carcinoma histologically). Very early onset ductal breast cancer is associated with *TP53* PGVs and therefore potentially explains the *TP53* PGV in this lobular case [29]. Given the extremely high odds ratios for *TP53* in very early onset bilateral and familial cases an increased risk of lobular cancer is nonetheless still possible.

Interestingly, an MS of ≥ 20 is usually associated with a probability of *BRCA1/2* involvement of around 35%; we only identified *BRCA1/2* PGVs in 11/75 (14.67%) in women with a MS ≥ 20 despite the MS already including a downward adjustment for LBC [8]. This almost certainly indicates that even the current reduction in score for lobular breast cancer of -2 is insufficient to reflect *BRCA1* risk. The absence of an association of LBC with *BRCA1* has already been noted by ourselves and others [6], with only 2/342 (0.58%) LBCs in *BRCA1* breast cancer patients in one study [6]. The higher odds ratio for *BRCA1* in our study is therefore likely to be explained by the strong family history of breast and/or ovarian cancer in relatives rather than a true association with *BRCA1*. Although the MS is specifically used for *BRCA1/2,* it does contain an element of assessing high risk for inherited breast cancer by scoring all breast cancers in the lineage and increasing the score in younger cases. Inclusion of the remaining genes only increased this to 20% with an identified PGV. The relative absence of family histories of ovarian cancers in the series (only 43/302 and 8/134 of those with panel testing) would suggest that the absence of PGVs in so many with high MS is likely due to missing heritability. This indicates there is still a greater proportion of the heritability unexplained for lobular cancer.

Although we have confirmed associations with well described breast cancer genes and lobular cancer, most of the ORs in our study and the literature are in keeping with the ORs for overall breast cancer risk, which does not suggest that these genes have a specific link to lobular cancers. In particular the OR of only 0.86 for c.1100delC in *CHEK2* is less than would be expected for ER + ductal cancer and does not support *CHEK2* being particularly associated with LBC. However, the ORs for *ATM* of 8.01 fold in this study and 3.50 fold from a previous study [23] if confirmed in further work would imply a more specific link to lobular cancers. The ORs for *BRCA1* in contrast were as high as expected for overall breast cancer risk; however this may reflect the rather low carrier frequency in the control population of only two of 1567 individuals, as well as the strong family histories of breast and/or ovarian cancer.

One of the limitations of this study was that we were unable to account for any copy number variations (CNVs) in samples tested through the BRIDGES study. Therefore, the results for some of the genes may be underestimated. For example, we have estimated that CNVs in *BRCA1* account for 20% of the PGVs in non-Jewish families [30]. We also did not have detailed pathology of the breast cancers in the families of those with familial breast cancer, although a number of families did have confirmed lobular family history, including the family with the *CDH1* variant. Also, as only a subset of the full cohort has been tested for the extended gene panel, there may be still undetected PGVs. In contrast to many other studies, we have been able to use local control samples rather than infer odds ratios from frequencies on gnomAD [31]. The relatively high frequency of *BRCA1/2* in PROCAS controls of 0.56% does not appear to be due to bias as 1^st^ degree breast cancer family history was not more frequent in subjects who provided a DNA sample. It is also very similar to a study of 50,726 adult biobank volunteers in the USA which found a 0.5% rate [32]. Another possible limitation is that there was no central pathology review. However, both of our *TP53*-positive cases were reviewed as part of their treatment at a major cancer centre (the Christie) and our study reflects actual practice, as pathologies are not usually reviewed in order to undertake genetic testing.

In summary, we have identified an association between *ATM* and an increased risk of LBC, but it is likely that further familial lobular cancer genes remain to be discovered as only 20% of patients with LBC and a family history of breast cancer with MS ≥ 20 were explained by currently known breast cancer predisposition genes.

## Supplementary Information

Below is the link to the electronic supplementary material.

Supplementary file1 (DOCX 76 kb)

## Data Availability

The datasets analysed during the current study are available from the corresponding author on reasonable request.
