# Supplementary material for: Extended gene panel testing in lobular breast cancer
Source: Fam Cancer. 2021 Mar 25;21(2):129–36. doi: 10.1007/s10689-021-00241-5 (PMC8964550; doi:10.1007/s10689-021-00241-5)
Supplement: Supplementary file 1 — Supplementary file1 (DOCX 76 kb) [file 10689_2021_241_MOESM1_ESM.docx]

Supplementary Figure 1: flowchart of the women tested for pathogenic variants in breast cancer associated genes.


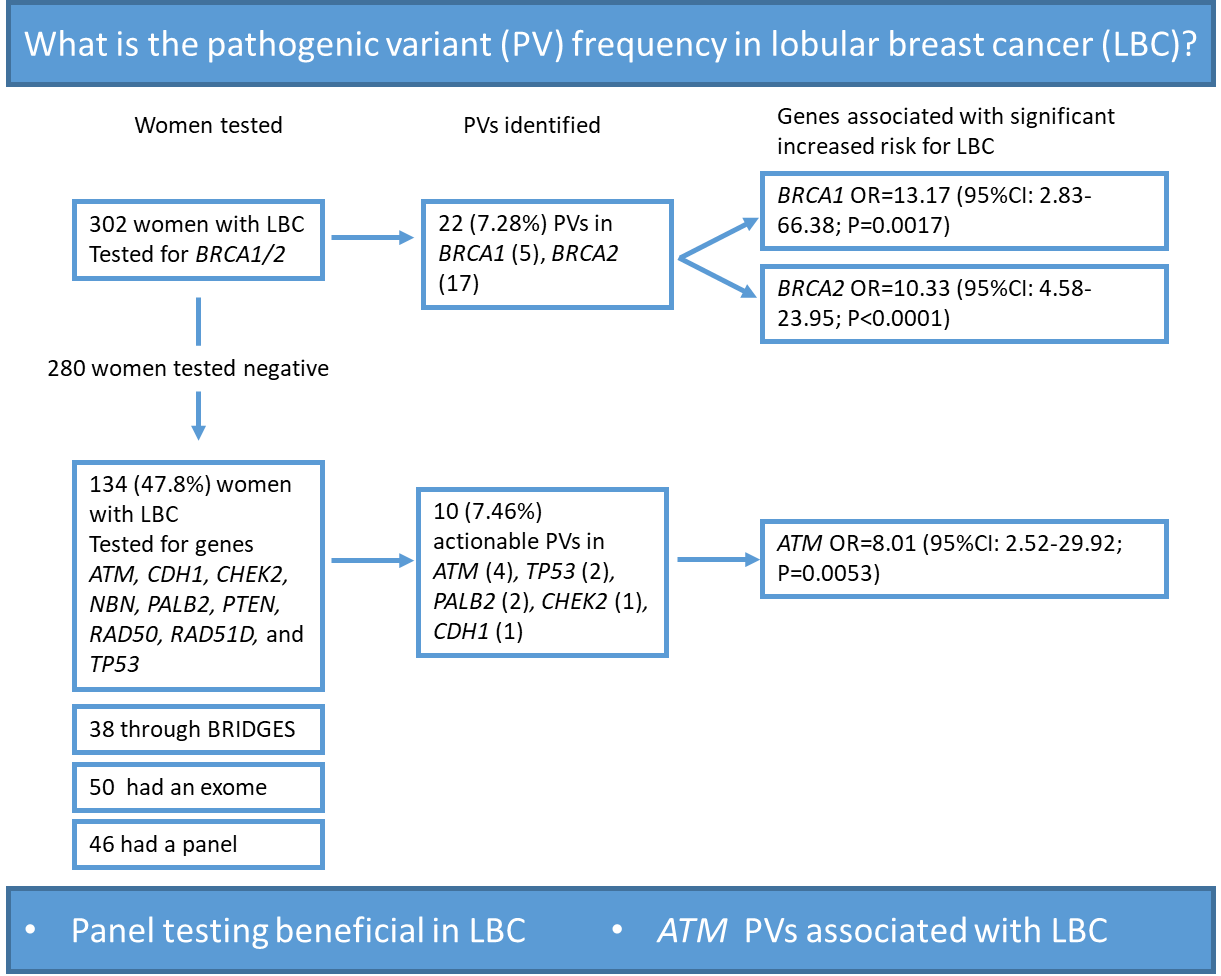
u
